# Osmotic Stress and ABA Affect Immune Response and Susceptibility of Grapevine Berries to Gray Mold by Priming Polyamine Accumulation

**DOI:** 10.3389/fpls.2018.01010

**Published:** 2018-07-11

**Authors:** Saloua Hatmi, Sandra Villaume, Patricia Trotel-Aziz, Essaid A. Barka, Christophe Clément, Aziz Aziz

**Affiliations:** Induced Resistance and Plant Bioprotection – RIBP EA 4707, SFR Condorcet FR-CNRS 3417, UFR Sciences, University of Reims, Reims, France

**Keywords:** *Botrytis cinerea*, immune response, osmotic stress, polyamines, *Vitis vinifera*

## Abstract

Abiotic factors inducing osmotic stress can affect plant immunity and resistance against pathogen attack. Although a number of studies have characterized grapevine responses to various forms of biotic and abiotic stresses, the relationships between osmotic stress response and susceptibility of mature berries to *Botrytis cinerea* still remain unknown. In this study, we investigated the effects of osmotic stress and abscisic acid (ABA) on defense responses of mature grapevine berries before and after *B. cinerea* infection. We focused on the possible involvement of polyamines in the interaction between osmotic stress response and susceptibility to *B. cinerea*. We showed that osmotic stress induced by PEG or sucrose, and exogenous ABA induce transient but low defense responses, including weak expression of *PR* genes and phytoalexin synthesis in mature berries. This was accompanied by an upregulation of *NCED2* involved in ABA biosynthesis and a large production of free polyamines. However, osmotic stress followed by *B. cinerea* infection primed berries for enhanced accumulation of polyamines, but slowed down the defense responses and increased susceptibility to the pathogen. A weak increase of diamine- and polyamine-oxidase activities was also recorded in stressed berries, but declined after pathogen infection. The pretreatment of stressed berries with appropriate inhibitors of diamine- and polyamine-oxidases further increased polyamine level and greatly lowered defense responses, leading to higher susceptibility to *B. cinerea*. These results suggest that increased polyamine titer through low activation of their oxidative degradation in grape berries may contribute at least in part to the weakening of defense responses and subsequent disease susceptibility.

## Introduction

Drought inducing osmotic stress is among the most serious abiotic constraints for global agriculture that may intensify during the next few decades (Zhao and Running, 2010). Emerging evidences suggest that abiotic stress can strongly modulate plant–pathogen interactions resulting in plant susceptibility or resistance to diseases (Sinha et al., 2016). These effects involve crosstalk between different responses during successive or combined biotic and abiotic stresses (Anderson et al., 2004; Atkinson et al., 2013; Seifi et al., 2013). Contrasting findings suggest that water deficit might differentially affect plant immunity and microbial pathogenesis in different pathosystems (Bidzinski et al., 2016; Sinha et al., 2016; Gupta et al., 2017). This type of stress can weaken plant immune responses or other metabolic pathways occurring during water stress then resulting in a predisposition of plants to pathogen infection. Recent evidences show that drought stress can suppress pathogen associated molecular pattern-triggered immunity and effector-triggered immunity in rice, which becomes highly susceptible to the blast fungus *Magnaporthe oryzae* infection (Bidzinski et al., 2016). The susceptibility could be attributed to increased levels of abscisic acid (ABA) in drought stressed plants which can interfere with pathogen-induced immune signaling pathways and thereby reduce the expression of defense-related genes (Fujita et al., 2006; Ton et al., 2009; Pieterse et al., 2012; Atkinson et al., 2013). However, successive abiotic and pathogen stresses may also lead to a priming state of plants for enhanced basal defenses resulting in plant resistance to pathogen. Some studies showed that drought-stressed Arabidopsis, chickpea, and tomato plants are more resistant to bacterial pathogens, *Pst* DC3000, *Pseudomonas syringae* pv. *phaseolicola*, and the fungus *Botrytis cinerea*, respectively (Achuo et al., 2006; Gupta et al., 2016; Sinha et al., 2016). Similarly, osmotic stress enhanced barley resistance to powdery mildew caused by *Blumeria graminis* through the primed formation of papillae (Wiese et al., 2004).

The overall response of plants to the successive or combined abiotic and biotic stress is generally governed by plant hormones, salicylate (SA), jasmonate (JA), and ethylene (ET) as key regulators of plant immunity (Pieterse et al., 2012). SA is generally required for plant defense against biotrophic pathogens, and JA and ET are considered to play their major role in plant defense against necrotrophic pathogens (Glazebrook, 2005; Pieterse et al., 2012). Furthermore, as for ABA, the SA and JA pathways are also reported to play a key role in mediating drought or salt tolerance and resistance to pathogens (Borsani et al., 2001; Seo et al., 2011; Ismail et al., 2012). Given their involvement in regulating both biotic and abiotic stress responses, a crosstalk between these signaling pathways has often been postulated. ABA can act as a negative regulator of defense responses mediated by SA and enhance plant susceptibility to pathogens (Mohr and Cahill, 2003). In addition to their antagonistic regulation, a positive crosstalk between SA and ABA pathways has also been reported. This synergistic interaction can result in stomatal closure contributing to drought tolerance (Miura et al., 2013).

Polyamines and their metabolic pathways constitute also integral parts of both adaptive responses to abiotic stress (Aziz et al., 1999; Bouchereau et al., 1999; Cuevas et al., 2008; Alcázar et al., 2010) and immune system upon plant–microbe interactions (Marina et al., 2008; Gonzalez et al., 2011; Nambeesan et al., 2012). Through their oxidative pathways, polyamines can generate oxidative burst mediating the hypersensitive response and the expression of defense genes (Cona et al., 2006; Yoda et al., 2006). Otherwise, polyamines were shown to interact with JA and ET signaling, thereby enhancing plant susceptibility to pathogens (Anderson et al., 2004; Nambeesan et al., 2012). Numerous biological functions have further been attributed to polyamines due in part to their polycationic nature, since they can interact with most of negatively charged molecules and stabilize their structure under various conditions (Alcázar et al., 2010). In plants, the major polyamines are putrescine, spermidine, and spermine. Putrescine can be synthesized from arginine or ornithine through arginine decarboxylase or ornithine decarboxylase, respectively (Bouchereau et al., 1999). Polyamine homeostasis involves also their oxidative degradation through copper amine oxidase (CuAO) and polyamine-oxidase (PAO). Some PAO isoforms are also involved in back-conversion of spermine to spermidine or putrescine (Moschou et al., 2008). These oxidative pathways may affect polyamine homeostasis and modulate hormone signaling and redox status, thereby conferring a protective role under stress conditions (Aziz et al., 1999; Moschou et al., 2008; Pál et al., 2015). Polyamine homeostasis can also be affected by an imbalance between activation of biosynthetic and oxidative pathways in stressed conditions, thus leading to an excess or deficiency of polyamines and conferring susceptibility to stress (Aziz et al., 1999; Nambeesan et al., 2012; Hatmi et al., 2015). This is consistent with the suggestion that the higher polyamine level is not always the better, but its regulatory role and the fine tuning of their level seem more important in stress responses (Pál et al., 2015).

Polyamine oxidation is also intrinsic to signaling mechanisms in both abiotic stress adaptation and disease resistance response (Cona et al., 2006; Moschou et al., 2008; Hatmi et al., 2014; Pal and Janda, 2017). The generation of H_2_O_2_ through the oxidative pathways of polyamines is intimately linked to improved resistance against pathogens and tolerance to abiotic stress (Takahashi et al., 2004; Cona et al., 2006; Moschou et al., 2008; Yoda et al., 2009). The generated H_2_O_2_ can induce the expression of various defense or stress responsive genes and inhibit pathogen growth. H_2_O_2_ is also involved in programmed cell death as well as in stomata opening (Yoda et al., 2006), and used in cross-linking and maturation of the cell wall by peroxidases (Takahashi et al., 2004; Cona et al., 2006; Yoda et al., 2009). Furthermore, polyamine pathways are connected to other signaling molecules such as nitric oxide, ET, and γ-aminobutyric acid (GABA) which are part of plant adaptation and immune response to abiotic stress and pathogen attack (Cona et al., 2006; Cuevas et al., 2008). It has been shown that the over accumulation of spermidine in transgenic tomato was associated to a weakened ET-induced defense responses, therefore increasing the susceptibility of fruit to *B. cinerea* (Nambeesan et al., 2012).

Although a number of studies have characterized grapevine responses to various forms of biotic and abiotic stresses, transcriptomic studies have revealed a weak expression of immune response in mature berries during early infection with *B. cinerea* (Bézier et al., 2002; Kelloniemi et al., 2015). More recently, it has been shown that osmotic stress attenuated defense responses in grapevine leaves after *B. cinerea* challenge and enhanced their susceptibility to the fungal pathogen (Hatmi et al., 2014). Other study using grapevine varieties with contrasting tolerance to drought stress illustrated that polyamine oxidation may affect the signaling network in grapevine leaves resulting in improved defense response (Hatmi et al., 2015). However, the relationships between osmotic stress response and susceptibility of mature grapevine berries to *B. cinerea* still remain unknown. The role of polyamines in the interaction between osmotic stress response and triggered immune defense after pathogen attack has not yet explored in grape berries.

The present study aimed to understand the relationships between osmotic stress and the susceptibility of mature grapevine berries to *B. cinerea*, and to unravel mechanisms mediating interactions between grapevine berry responses to osmotic stress and pathogen infection. Here, we first investigated the effects of osmotic stress and ABA on defense responses of detached berries before and after *B. cinerea* inoculation. We further focused on the role of polyamines in the interaction between osmotic stress response and immune response. We also used pharmacological approach to assess whether oxidative pathways of polyamines may affect defense responses induced by osmotic stress and the susceptibility of berries to *B. cinerea*.

## Materials and Methods

### Plant Material and Stress Treatments

Grapevine berries (*Vitis vinifera* cv. Chardonnay) were collected from vineyards at full ripening. Detached berries with pedicels were washed three times with sterilized water and the pedicels were dipped in 12.5 mM MES buffer containing 7.5 mM KCl and 5 mM CaCl_2_, pH 6 as a control medium. Osmotic stress was applied using 400 g L^-1^ PEG 6000 or 600 mM sucrose to the control medium. ABA was also added to the control medium at 100 μM in 0.02% ethanol. ABA concentration used in this study approximates ABA concentration of berries prior to the onset of ripening (Giribaldi et al., 2010). Samples were put in growth chamber with a 16/8 h photoperiod, at a photosynthetic photon flux density of 60 μmol m^-2^ s^-1^ and a 25°C/22°C day/night, respectively. Berries were harvested at 0, 3, 7, and 12 days of osmotic stress or ABA treatment. On each sampling time berries were deseeded, frozen, and ground to fine powder in liquid nitrogen. Three replicates were used from independent batches of 12–15 berries.

For the priming state, berries were first treated by dipping the pedicels in control medium supplemented with 400 g L^-1^ PEG 6000 or 600 mM sucrose or 100 μM ABA for 72 h, then single berries were drop-inoculated with *B. cinerea* and harvested at 7 days post-infection (dpi).

### Inhibitor Treatments

Two pharmacological chemicals were used in this study, aminoguanidine (AG, Sigma) and guazatine (GUA, Sigma) as specific and competitive inhibitors of CuAO (Xing et al., 2007) and PAO, respectively (Yoda et al., 2006). Single berries were pretreated at the level of the pedicel with 2 mM AG or 100 μM GUA for 12 h, then transferred to control or stressing medium with 400 g L^-1^ PEG 6000 or 600 mM sucrose for 3 days under the same conditions described above. Berries were then used for analysis of defense- or stress-responsive genes and metabolites before and after pathogen infection.

### Fungal Inoculation and Disease Assay

Fungal culture and preparation of conidial spores were as described previously (Aziz et al., 2003). After pretreatment of berries with inhibitors or with osmotic agents for 3 days, one needle prick wound was applied in each single berry and covered with 5 μL of a conidial suspension of *B. cinerea* (5 × 10^5^ conidia mL^-1^). Disease symptoms were measured on 20–25 berries from three independent experiments at 7 or 10 days post-inoculation. Disease rating was assessed as the fraction of berries falling in different classes: (I) spreading lesion with less than the 20% of the berry surface; (II) spreading lesion with the 21–35% of the berry surface; (III) spreading lesion with the 36–50% of the berry surface; (IV) spreading lesion with more than 50% of the berry surface.

### Polyamine Analysis

Berries were sampled at different times of treatment, frozen and powdered with liquid nitrogen. Free polyamines were extracted with cold 1 M HCl (2:1, w/v) on ice, as described by Aziz et al. (2001). The homogenate were kept for 1 h at 4°C and then centrifuged for 20 min at 24,000 *g*. The supernatants were used for the dansylation. The aliquots of 250 μL from the supernatants were mixed with sodium carbonate and dansyl chloride as described in Hatmi et al. (2014). The mixture was incubated at room temperature for 16 h. The reaction was stopped with 300 μL of proline solution (100 mg mL^-1^). Derivatized polyamines were extracted into 2 mL of ethyl acetate. After the organic phase was evaporated under nitrogen stream, the residue was solubilized with 1 mL of methanol, filtered through 0.22 μm PTFE filters and dansyl polyamines were analyzed using Acquity UPLC system (Waters), and a BEH C18, 1.7 μm, 2.1 mm × 100 mm column heated at 30°C. Dansyl polyamines were eluted with acetonitrile: water solvent gradient and detected by an Acquity fluorimeter (Waters) with an excitation wavelength of 365 nm and an emission wavelength of 510 nm as described by Hatmi et al. (2014). Polyamines were quantified after calibration with external standards (Sigma).

### Phytoalexin Analysis

Stilbenic phytoalexins were extracted from 1.5 g of freeze-dried powder with 2 mL of methanol 100% in the dark at room temperature. Tubes were placed in shaker for 1 h and then centrifuged for 10 min at 8000 *g* (Hatmi et al., 2014). The supernatants were dried under vacuum and residues were solubilized with 1 mL of methanol then filtered through 0.22 μm PTFE filters. Resveratrol and ε-viniferin were analyzed using an Acquity UPLC system (Waters, Milford, United States) with an Acquity UPLC BEH C18, 1.7 μm, 2.1 mm × 100 mm, heated at 40°C and a gradient from 10 to 90% acetonitrile as described in Hatmi et al. (2014). Phytoalexins were detected with an Acquity fluorimeter (Waters) at an excitation wavelength of 330 nm with an emission wavelength of 375 nm. Resveratrol and ε-viniferin were identified and quantified with reference of retention time and calibration with external standards.

### RNA Extraction and qRT-PCR

Total RNA was isolated from deseeded berries using the Extract-All reagent (Eurobio) and 150 ng was used for reverse-transcription using the Verso cDNA Synthesis kit (Thermo Electron) according to the manufacturer’s instructions. The transcript levels were determined by real-time PCR using the CFX96 system (Bio-Rad) and absolute blue qPCR SYBR Green as recommended by the manufacturer (Thermo Electron). PCRs were performed using a 10-fold cDNA dilution in duplicates as template in 96-well plates in a 15-μL final volume containing 1× SYBR Green I mix (including Taq polymerase, dNTPs, SYBR Green dye) and 280 nM forward and reverse primers. Cycling parameters were 15 min of Taq polymerase activation at 95°C, followed by 40 two-step cycles composed of 10 s of denaturation at 95°C and 45 s of annealing and elongation at 60°C. The EF1 gene was used as a reference gene and experiments were repeated three times. The specific primers of analyzed genes are listed in Supplementary Table S1. Relative gene expression was determined with the formula fold induction: 2^-ΔΔCt^, where ΔΔCt = [*Ct* TG (US) -*Ct* RG (US)] - [*Ct* TG (RS) -*Ct* RG (RS)], where Ct is cycle threshold, Ct value is based on the threshold crossing point of individual fluorescence traces of each sample, TG is target gene, RG is reference gene, US is unknown sample, and RS is reference sample. Integration of the formula was performed by the CFX Manager 3.0 software (Bio-Rad). The reference sample is the control sample at each sampling time point chosen to represent 1× expression of the target gene.

### Amine-Oxidase Activity

Copper amine oxidase and PAO assays were performed as described in Hatmi et al. (2015). Briefly, enzymes were extracted on ice from 250 mg of powdered plant material with 1 mL of 100 mM potassium phosphate buffer, pH 6.5 for PAO or pH 7.0 for CuAO. Extracts were centrifuged at 12,000 *g* and 4°C for 10 min, then CuAO and PAO activity was measured with spectrophotometer using 2 mM putrescine or 2 mM spermidine as the substrates. The formation of pink adduct resulting from the oxidation and condensation of 4-aminoantipyrine and 3,5-dichloro-2-hydroxybenzenesulfonic acid (Sigma-Aldrich) catalyzed by horseradish peroxidase (Cona et al., 2006) was quantified (ε515 = 2.6 × 104 M^-1^ cm^-1^) at 25°C.

### Statistical Analysis

Data are averages of three independent experiments and are shown as means ± SDs. Statistical analyses were carried out using the SigmaStat 3.5 software. Data were analyzed using two-way analysis of variance (ANOVA). For treatment effect, mean values were compared by Tukey’s test (*P* < 0.05).

## Results

### Osmotic Stress and ABA Induce Transient, but Low Defense Responses in Berries

**FIGURE 1:**
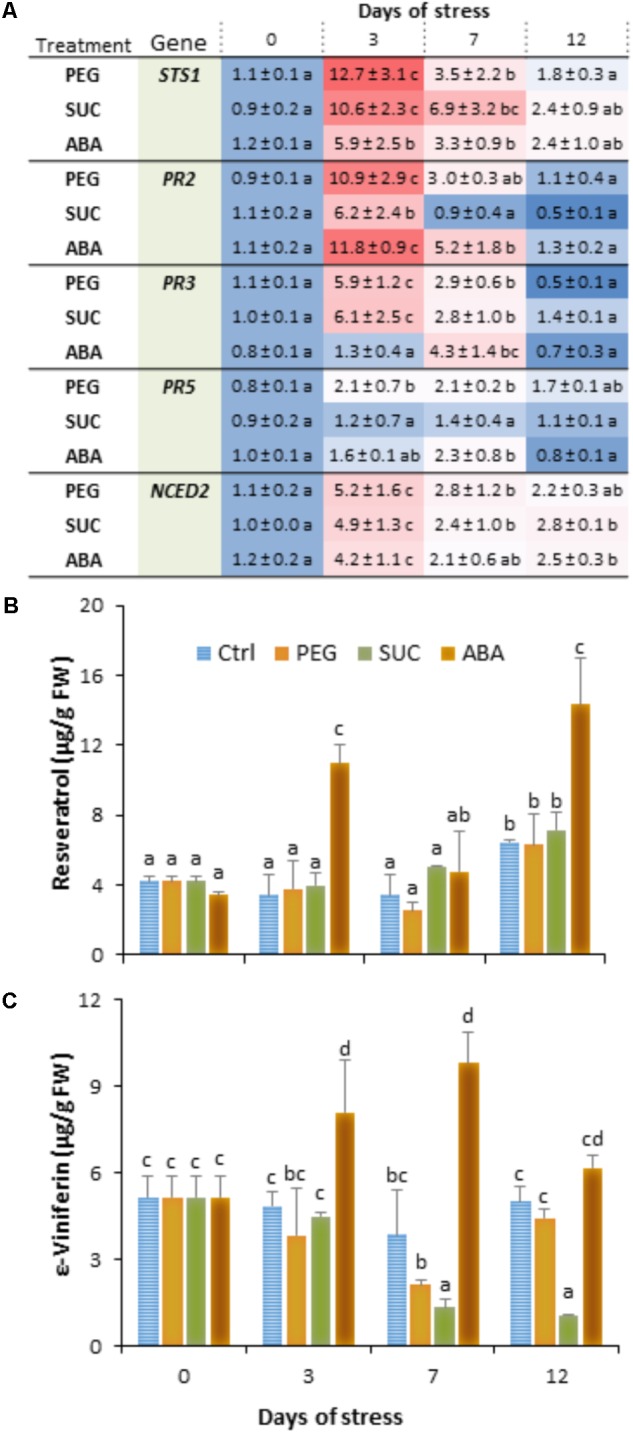
Expression of defense- or stress-related genes and phytoalexin accumulation in grapevine berries in response to osmotic stress and ABA treatment. Single berries were treated by immersing the pedicel in control medium (Ctrl) containing 400 g L^-1^ PEG 6000 (PEG) or 600 mM sucrose (SUC) or 100 μM ABA and harvested at the indicated times. Values of gene expression **(A)** are expressed as the fold increase in transcript level relative to the corresponding control at each sampling time. *trans*-Resveratrol **(B)** and ε-viniferin **(C)** concentrations were determined in control, osmotic stressed- and ABA-treated berries. Data are means ± SD representative of three experiments. Different letters indicate significant differences (Duncan’s multiple range test, *P* < 0.05).

We investigated whether exposure of detached berries to PEG or sucrose inducing osmotic stress or ABA treatment can affect immune response. Using quantitative real-time PCR (qRT-PCR) and specific primers (Supplementary Table S1), we examined the expression levels of some defense-related genes, including *STS1* (a stilbene synthase), *PR2* (a β-1,3-glucanase), *PR3* (an acidic chitinase IV), and *PR5* (a thaumatin-like protein), as well as *NCED2* encoding 9-*cis*-epoxycarotenoid dioxygenase involved in ABA biosynthesis. Berries were collected at different time points after PEG, sucrose, or ABA treatment. We showed that most of the targeted genes were transiently upregulated in berries during osmotic stress or ABA treatment (**Figure 1A**). The steady state levels of mRNA for *STS1*, *PR2*, and *PR3* moderately increased at day 3 in response to osmotic stress and ABA compared to control. The highest expression of *STS1* (12-fold) and *PR3* (6-fold) was observed in response to PEG and sucrose, while *PR2* was more expressed with PEG (11-fold) and ABA (12-fold). The transcript levels were subsequently returned to their basal values after 12 days of osmotic stress and ABA treatment. The expression of *PR5* gene is only slightly increased at 3 or 7 days post-treatment with PEG and ABA. The expression of *NCED2* was also transiently upregulated in berries, albeit to much lower extent during the exposure to both osmotic stress and ABA (**Figure 1A**). The expression being maximal at day 3, it reached 5.2-fold, compared with control.

We also analyzed the main stilbenic phytoalexins, *trans*-resveratrol (*trans*-3,5,4′-trihydroxy-*trans*-stilbene) and its dimer *trans*-ε-viniferin as one of the most important responses of basal and induced immunity. As expected, ripe berries showed high basal levels of *trans*-resveratrol (**Figure 1B**) and *trans*-ε-viniferin (**Figure 1C**) in control conditions. However, when they were exposed to osmotic stress or ABA, berries exhibited different responses with respect to stilbene accumulation. The amount of resveratrol did not change during exposure of berries to PEG and sucrose compared to control (**Figure 1B**). However, resveratrol level increased in ABA-treated berries by about 2.5- to 3.5-fold at days 3 and 12. ε-Viniferin was also present to similar extents compared to resveratrol in control berries (**Figure 1C**). However, its amounts significantly lowered in response to osmotic stress, and the decrease was prominent with sucrose than with PEG at days 7 and 12. Again, as for resveratrol, ε-viniferin amount increased albeit to a much lower level in response to ABA, and peaked at day 3 and 7 of treatment.

### Osmotic Stress and ABA Induce Changes in Free Polyamine Titers in Berries

**FIGURE 2:**
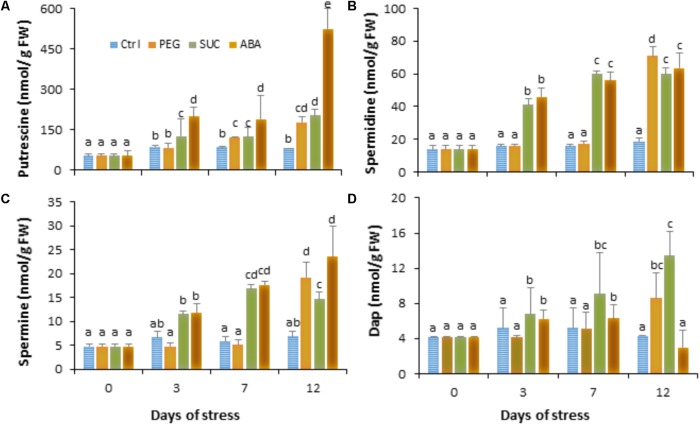
Free polyamine levels in single berries in response to osmotic stress and ABA treatment. Detached berries were treated by dipping the pedicel in control medium (Ctrl) amended with 400 g L^-1^ PEG 6000 (PEG) or 600 mM sucrose (SUC), or 100 μM ABA and harvested at the indicated times. Putrescine **(A)**, spermidine **(B)**, spermine **(C)**, and 1,3-diaminopropane **(D)** concentrations were determined in control, osmotic stressed and ABA-treated berries. Values are means ± SD of three experiments. Different letters indicate significant differences (Duncan’s multiple range test, *P* < 0.05).

To investigate the role of polyamines in berries following exposure to stress, we first quantified the diamine putrescine, the polyamines spermidine and spermine and their oxidation product 1,3-diaminopropane (Dap) during osmotic stress or ABA treatment. Data (**Figure 2**) showed that putrescine (**Figure 2A**) and spermidine (**Figure 2B**) are the most abundant polyamines in ripe berries. The amounts of polyamines did not change in PEG-treated berries during the first days, while they significantly increased from the third day in both sucrose- and ABA-treated berries (**Figure 2**). Thereafter, the level of the four polyamines became important after a 12 days in response to osmotic stress and ABA, excepted for Dap which significantly decreased in ABA-treated berries (**Figure 2D**). The putrescine content increased by about two- to threefold and those of spermidine and spermine increased by about three to five times in the osmotically stressed and ABA-treated berries. However, the amount of Dap slightly increased in response to PEG and sucrose, but remained unchanged at 12 days in ABA-treated berries. These results suggest that both osmotic stress and ABA treatment upregulate polyamine biosynthetic pathways over than their oxidation.

### Osmotic Stress Potentiate Free Polyamine Accumulation but Not CuAO and PAO Activity After Pathogen Infection

**FIGURE 3:**
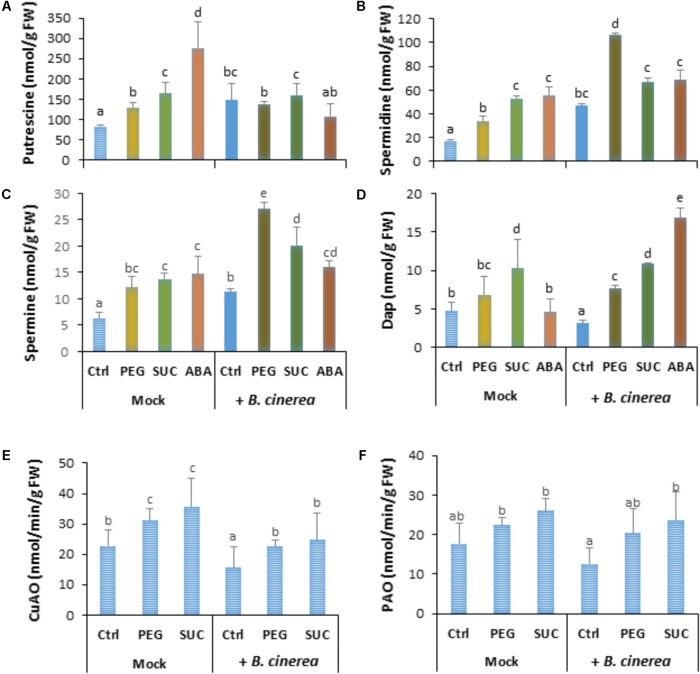
Polyamine accumulation and CuAO and PAO activity in osmotically stressed or ABA-treated grapevine berries after *B. cinerea* infection. Berries were first treated by dipping the pedicel in control medium (Ctrl) supplemented with 400 g L^-1^ PEG 6000 (PEG) or 600 mM sucrose (SUC) or 100 μM ABA for 72 h, then drop-inoculated with *B. cinerea* and harvested at 7 dpi. Values of putrescine **(A)**, spermidine **(B)**, spermine **(C)**, 1,3-diaminopropane **(D)**, and activity of CuAO **(E)** and PAO **(F)** are means ± SD of three experiments. Different letters indicate significant differences (Duncan’s multiple range test, *P* < 0.05).

In order to investigate if the induced change in free polyamines and defense responses by osmotic stress or ABA are influenced by *B. cinerea* infection, berries were first exposed to osmotic stress or ABA for 3 days then inoculated with *B. cinerea*. Data showed that infection of control berries with *B. cinerea* resulted in enhanced level of putrescine (**Figure 3A**), spermidine (**Figure 3B**), and spermine (**Figure 3C**), while the amount of Dap decreased (**Figure 3D**). In most cases, pretreatment of detached berries with osmotic agents maintained putrescine (**Figure 3A**) to a higher level after pathogen challenge, while the amounts of spermidine (**Figure 3B**), spermine (**Figure 3C**), and Dap (**Figure 3D**) were significantly increased compared to non-infected or control infected berries. Similarly, in berries pretreated with ABA then challenged with *B. cinerea* the level of putrescine remained unchanged, whereas spermidine and spermine contents increased, but to a lower extent compared to osmotically stress berries. In the same condition Dap content increased after pathogen infection. This suggests that both osmotic stress and ABA potentiate free polyamine accumulation probably by activating their biosynthesis more than their oxidation after *B. cinerea* infection.

Copper amine oxidase and PAO activity was also assayed to determine whether polyamine levels could be related to extent of their oxidative degradation in osmotically stressed berries after pathogen challenge. Data showed that CuAO (**Figure 3E**) activity was slightly enhanced by osmotic stress (by about 1.2-fold in mock berries), while the effects of *B. cinerea* infection resulted in a significant reduction of CuAO activity in both control, and stressed berries. Similarly, PAO activity (**Figure 3F**) was stimulated to a lower (but not significant) extent in PEG- and sucrose-stressed berries. However, no clear difference of PAO activity was observed between mock and *B. cinerea*-infected berries. The low or reduced activity of CuAO and PAO in infected berries is consistent with the primed accumulation of free polyamines after *B. cinerea* challenge.

### Osmotic Stress and ABA Slow Down Phytoalexin Accumulation After Infection and Increase Susceptibility to *B. cinerea*

**FIGURE 4:**
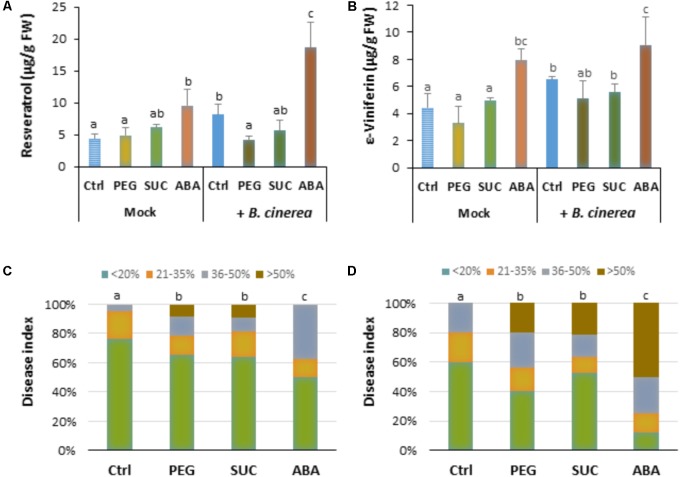
Phytoalexin accumulation and susceptibility of osmotically stressed or ABA-treated grapevine berries to *B. cinerea*. Berries were first treated by dipping the pedicel in control medium supplemented with 400 g L^-1^ PEG 6000 (PEG), 600 mM sucrose (SUC) or 100 μM ABA for 72 h, then drop-inoculated with *B. cinerea* and harvested at 7 dpi. Values of resveratrol **(A)** and ε-viniferin **(B)** are means ± SD of three experiments. Disease rating was evaluated at 7 **(C)** and 10 days post-inoculation **(D)** as the fraction of berries falling into the following classes: (I) spreading lesion of less than 20% of the berry area; (II) spreading lesion of 21–35% of the berry area; (III) spreading lesion of 36–50% of the berry area; (IV) spreading lesion of more than 50% of the berry area. Values are means of the average disease index from 20 to 30 berries from three batches. Different letters indicate significant differences (Duncan’s multiple range test, *P* < 0.05).

To determine whether osmotic stress or exogenous ABA can affect defense responses after *B. cinerea* infection, the main stilbenic phytoalexins were analyzed in both mock and infected berries. As shown previously, the amounts of phytoalexins, resveratrol (**Figure 4A**) and ε-viniferin (**Figure 4B**) in osmotically stressed berries (mock) were comparable to that of control, while the amount of both stilbenes increased in response to ABA (**Figure 4**). In *B. cinerea*-infected control the amount of resveratrol and ε-viniferin increased (**Figures 4A,B**). However, the amounts of phytoalexins was not primed by PEG or sucrose after pathogen infection (**Figures 4A,B**), and only a potentiated accumulation of resveratrol, but not of ε-viniferin was observed with ABA after infection (**Figure 4A**). Our data indicate that osmotic stress has no consistent priming effect on phytoalexin accumulation in berries after pathogen challenge.

Disease incidence experiments were conducted to determine whether the effects of osmotic stress or ABA benefit *B. cinerea* development in mature berries. Seven days after infection, large control berries developed necrosis with less than 20% (**Figure 4C**). Pretreatment of berries with PEG or with sucrose significantly enhanced disease incidence caused by the pathogen. Within 7 dpi, approximately 40% of osmotically stressed berries were infected at more than 35% (**Figure 4C**). Similarly, more than 50% of ABA-treated berries showed heavy gray mold symptoms (**Figure 4C**). After 10 dpi, osmotically stressed berries showed a dramatic increase of gray mold disease (**Figure 4D**), with approximately 60 and 50% of berries heavily infected with PEG and sucrose, respectively. In the case of ABA, the number of heavily infected berries increased and reached more than 75% of the berry area.

### Inhibitors of Polyamine Oxidation Strongly Increase Polyamine Levels, and Reduce Defense Responses in Osmotically Stressed Berries

**FIGURE 5:**
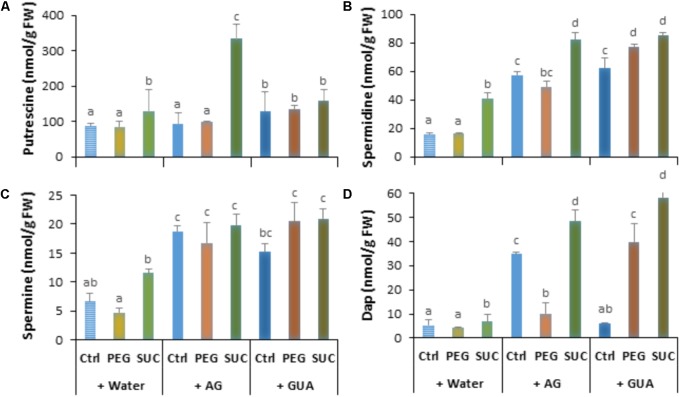
Free polyamine levels in osmotically stressed detached berries pretreated with aminoguanidine (AG) or guazatine (GUA) as inhibitors of CuAO and PAO. Detached berries were pretreated at the level of the pedicel with water, 2 mM AG or 100 μM GUA for 12 h, then transferred to a new medium containing 400 g L^-1^ PEG 6000 (PEG) or 600 mM sucrose (SUC), and harvested at day 3 post-stress. Values of putrescine **(A)**, spermidine **(B)**, spermine **(C)**, and 1,3-diaminopropane **(D)** are means ± SD of three experiments. Different letters indicate significant differences (Duncan’s multiple range test, *P* < 0.05).

The above reported data indicated that osmotic stress and ABA increased polyamine levels, and susceptibility of grapevine berries to *B. cinerea*, but poorly induced some defense responses. We hypothesized that increased amounts of polyamines may be linked at least in part to low activation of polyamine oxidation during osmotic stress or pathogen infection. To assess whether oxidative pathways of polyamines may affect defense responses induced by osmotic stress and the susceptibility of berries to *B. cinerea* we used two inhibitors; AG and GUA as specific and competitive inhibitors of CuAO and PAO, respectively. The effect of AG and GUA was first evaluated on the polyamine levels in the osmotically stressed berries. As shown in **Figure 5**, although putrescine content showed no significant differences between control and pretreated berries with AG (**Figure 5A**), the amounts of spermidine (**Figure 5B**), spermine (**Figure 5C**), and Dap (**Figure 5D**) increased significantly with AG. The AG effect resulted also in enhanced levels of spermidine, spermine, and Dap in osmotically stressed berries. This effect was more apparent with sucrose than PEG. GUA, however, slightly increased the level of putrescine (**Figure 5A**) in control berries, but strongly enhanced the levels of spermidine (**Figure 5B**) and spermine (**Figure 5C**). In the same conditions Dap content decreased by about twofold compared to the control (**Figure 5D**). The contents of the four polyamines also increased in PEG- and sucrose-stressed berries pretreated with GUA. The CuAO and PAO activity was also examined in the presence of AG and GUA. It was shown that both inhibitors reduced the enzymatic activity over than 50 and 60% in both control and stressed berries (data not shown). These results emphasize the contribution of the CuAO and PAO pathways in polyamine homeostasis in both control and stressed conditions.

**FIGURE 6:**
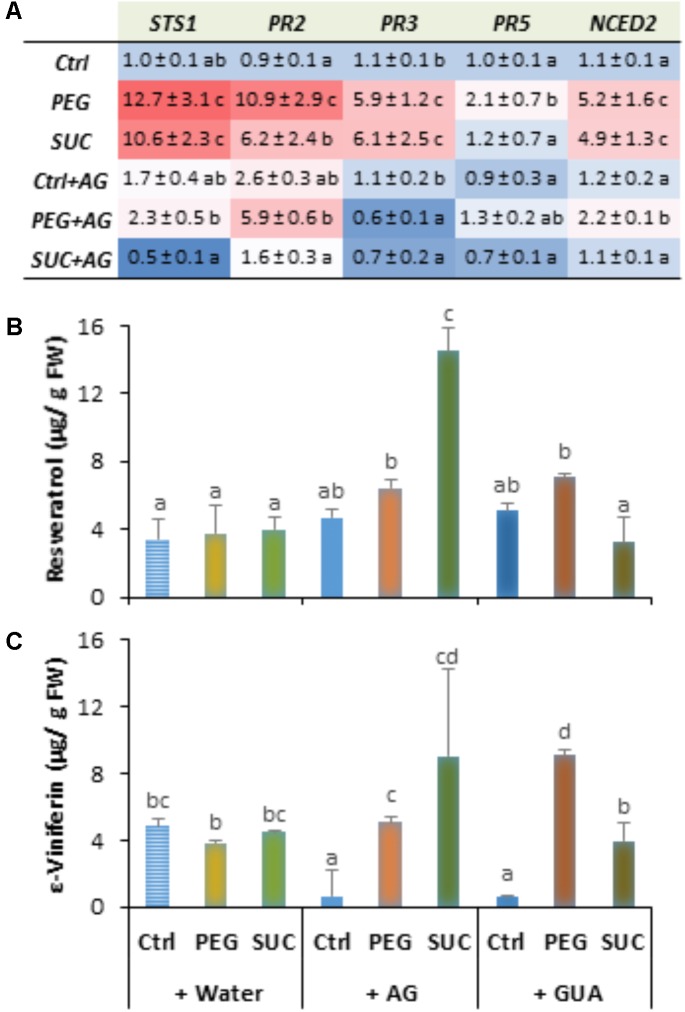
Expression of defense- or stress-related genes and phytoalexin accumulation in osmotically stressed grapevine berries pretreated with aminoguanidine (AG), or guazatine (GUA) as inhibitors of CuAO and PAO, respectively. Detached berries were pretreated at the pedicel level with water, 2 mM AG or 100 μM GUA for 12 h, then transferred to a new medium containing 400 g L^-1^ PEG 6000 (PEG) or 600 mM sucrose (SUC), and harvested at day 3 post-stress. Gene expression **(A)** is expressed as the fold increase in transcript level relative to the corresponding control at 3 days post-stress. *trans*-Resveratrol **(B)** and ε-viniferin **(C)**. Data are means ± SD representative of three experiments. Different letters indicate significant differences (Duncan’s multiple range test, *P* < 0.05).

The steady state level of defense responses was then examined in the presence of inhibitors. Data (**Figure 6A**) showed that AG without osmotic stress did not affect the basal level of defense gene transcripts, except for *PR2* which increased by about 2.6-fold compared to control. However, AG treatment followed by osmotic stress strongly lowered the osmo-induced expression of all studied genes. The expression of *STS*, *PR3*, and *PR5* was even repressed to a lower level with AG after sucrose stress. Similar results were obtained with GUA (data not shown). The expression of *NCED2* involved in ABA synthesis followed the same trends after AG and/or osmotic stress application (**Figure 6A**). Data support the idea that polyamine oxidation may play a critical role in immune response, involving ABA synthesis under osmotic stress condition.

Similarly, AG or GUA alone did not affect the production of resveratrol (**Figure 6B**), but significantly reduced the ε-viniferin level in control berries (**Figure 6C**). However, the AG-treated berries exhibited a significant accumulation of both resveratrol and ε-viniferin after osmotic stress, especially with sucrose. While GUA had only a slight effect on phytoalexin content of sucrose-stressed berries, those stress with PEG showed only enhanced level of ε-viniferin, suggesting that phytoalexin response was at least partly under the control of polyamine oxidation under osmotic stress.

### Inhibitors of Polyamine Oxidation Increase the Susceptibility of Osmotically Stressed Berries to *B. cinerea*

**FIGURE 7:**
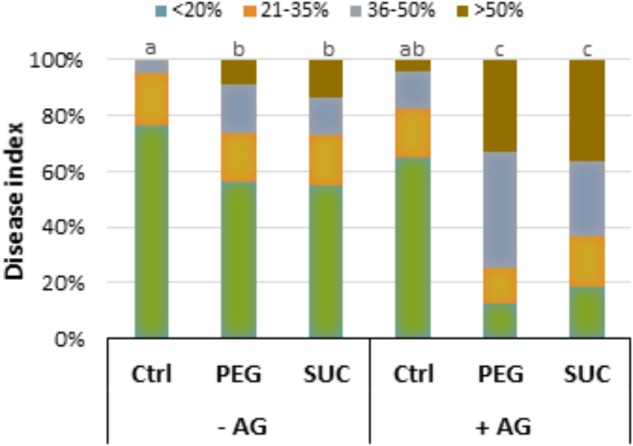
Effect of aminoguanidine (AG), an inhibitor of CuAO, on the osmotic stress-induced susceptibility of grapevine berries to *B. cinerea*. Detached berries were pretreated at the pedicel level with water (–AG) or 2 mM AG (+AG) for 12 h, then transferred to a new medium containing 400 g L^-1^ PEG 6000 (PEG) or 600 mM sucrose (SUC) for 72 h, and inoculated with *B. cinerea*. Disease rating was evaluated at 7 days post-inoculation as the fraction of berries falling into the following classes: (I) spreading lesion of less than 20% of the berry area; (II) spreading lesion of 21–35% of the berry area; (III) spreading lesion of 36–50% of the berry area; (IV) spreading lesion of more than 50% of the berry area. Values are means of the average disease index from 20 to 30 berries from three batches. Different letters indicate significant differences (Duncan’s multiple range test, *P* < 0.05).

The relationship between polyamine oxidation and the susceptibility to *B. cinerea* was then examined by treating berries with AG or GUA prior exposure to sucrose- or PEG-induced osmotic stress and pathogen infection. The percentage of diseased berries was determined at 7 dpi. As shown previously, osmotically stressed berries were highly susceptible to *B. cinerea*, and showed more extensive necrosis than non-stressed ones (**Figure 7**). Pretreatment of berries with AG followed by osmotic stress resulted in a marked amplification of gray mold symptoms with a large proportion of berries heavily infected (**Figure 7**). The disease rating was dramatically increased with approximately more than 60 and 75% of berries heavily infected beyond 35% after sucrose and PEG stress, respectively. Similar results were obtained when berries were pretreated with GUA prior to osmotic stress (data not shown). In the same conditions, levels of polyamines were greatly increased, suggesting that the weakened polyamine oxidation in mature berries under osmotic stress can greatly enhance their susceptibility to the necrotrophic fungus *B. cinerea*.

## Discussion

Grapevine (*V. vinifera*) varieties are highly susceptible to various environmental factors and pathogenic fungi, especially at the ripening stages. Up to now research on grapevine has focused on responses to individual stresses, but the interactions between abiotic and biotic stresses and their impact on susceptibility or resistance to pathogens still remain unknown. Therefore, understanding the mechanisms involved in adaptive response of the grapevine berries and its cross-link with the susceptibility to gray mold is of great interest for the future sustainable viticulture. We previously reported that osmotic stress or ABA attenuated defense responses triggered by *B. cinerea* in grapevine leaves (Hatmi et al., 2014). A close connection between water stress tolerance and the ability of grapevine cultivars to express high defense responses in leaves and then to resist better to the pathogen *B. cinerea* was also reported (Hatmi et al., 2015). These reports emphasize the importance of ABA and polyamine homeostasis in grapevine leaves under osmotic stress as a part of the host-signaling network that affects immune response and disease susceptibility. Using ripe berries, we showed that osmotic stress and ABA potentiate polyamine accumulation but slow down defense responses in ripe berries, especially after *B. cinerea* infection. Pharmacological experiments also provided evidence that inhibition of polyamine oxidation greatly reduced the expression of *PR* genes and *NCED2*, a key gene involved in ABA biosynthesis, and to lesser extent phytoalexin accumulation in osmotically stressed berries.

### Osmotic Stress and ABA Induce a Low Expression of Defense Responses in Grapevine Berries

We showed that both osmotic stress and ABA induced a transient but low expression of *PR2* and *PR3* genes, which were responsive to SA in grapevine (Dufour et al., 2013), and *STS1* gene encoding a stilbene synthase, as well as a low accumulation of resveratrol and ε-viniferin. However, the expression of *PR5* encoding a thaumatin-like protein present I a large quantity in mature berries (Monteiro et al., 2003), remained unchanged. This emphasizes the importance of osmotic status in regulating immune response in ripe berries. Osmotic stress and ABA also increased the transcript level of *NCED2* involved in ABA biosynthesis. This is in accordance with other studies showing that exogenous ABA triggered its own biosynthesis and increased ABA concentration in grapevine berries (Giribaldi et al., 2010). This also suggests that, apart from its role in mediating plant responses to osmotic stress, ABA may also have an important role in regulating defense responses in ripened berries and under abiotic stress. It has been shown that ABA can negatively regulate the production of phytoalexins that play a role in plant defense in various plants, and interact with JA, ET, and SA signaling pathways (Audenaert et al., 2002; Anderson et al., 2004) as well as with polyamine metabolism (Alcázar et al., 2010). All these compounds have been associated with plant immune response to pathogens (Glazebrook, 2005; Ton et al., 2009; Hatmi et al., 2014).

### Osmotic Stress and ABA Induce Polyamine Accumulation in Grapevine Berries

In the present work, we provide evidence that both osmotic stress and ABA play a key role in the polyamine homeostasis in ripe berries. We showed that the levels of free polyamines greatly enhanced after exposure to osmotic stress induced by PEG or sucrose, or to ABA. The concentration of 1,3-Dap, as an oxidation product of spermidine and spermine, slightly increased in response to PEG and sucrose, but significantly decreased in ABA-treated berries. The level of conjugated polyamines remained unchanged under osmotic stress in ripe berries (data not shown). Despite differential responses of polyamines, these results suggest that both osmotic stress and ABA induced free polyamine accumulation probably by activating their biosynthesis more than their oxidation, which seems poorly activated in stressed berries. However, we cannot exclude the possibility that the levels of polyamines in berries can be regulated by the back-conversion through PAO (Moschou et al., 2008). Similar effects have been observed in *B. cinerea*-infected berries which showed a significant increase of free polyamine titers. This fits well with the observation that polyamine oxidation through CuAO and PAO may play a regulatory role in abiotic stress and immune response (Cona et al., 2006; Yoda et al., 2006; Hatmi et al., 2015). Polyamine oxidation can generate H_2_O_2_ as a signal molecule in plant tissue mediating the hypersensitive response and the expression of defense genes (Cona et al., 2006; Yoda et al., 2006; Pál et al., 2015). Polyamine pathways are also linked to other important signaling pathways involved in either abiotic stress or disease tolerance, including ET, GABA, nitric oxide, and ABA (Cona et al., 2006; Cuevas et al., 2008).

### Osmotic Stress and ABA Potentiate Polyamine Accumulation but Weaken Defense Responses After Infection With *B. cinerea*

We also showed that osmotic stress followed by *B. cinerea* infection primed spermidine and spermine accumulation, but lowered the production of phytoalexins in berries. The potentiated polyamine level could be related to an increase of polyamine synthesis and/or low activation of their oxidation after pathogen challenge. In this study, a weak increase of diamine- and polyamine-oxidase activities was recorded in stressed berries, but declined after pathogen infection. This is consistent with increased level of polyamines in stressed berries after pathogen infection. The potentiated accumulation of polyamines in berries is associated with the weak expression of defense responses after infection, and enhanced susceptibility to *B. cinerea*. These results suggest that a high accumulation of polyamines in osmotically stressed or ABA-treated berries may not be in favor for berries to resist to the necrotrophic fungus. This emphasizes the importance of polyamine level as a critical regulator of both biotic and abiotic stress responses. Accumulation of polyamines can be toxic to berries, while their catabolism through CuAO and PAO could contribute to the adjustment of polyamine levels and thus to disease resistance. It is so conceivable that ABA-induced osmotic stress could be a primary factor predisposing grapevine berries to gray mold (Hatmi et al., 2014). It has also been shown that drought stress or ABA treatment can increase the susceptibility of Arabidopsis to an avirulent strain of *P. syringae*, while in tomato ABA increased susceptibility to *B. cinerea* and *Erwinia chrysanthemi* (Audenaert et al., 2002; Mohr and Cahill, 2003; Seifi et al., 2013). By contrast, ABA has been shown to be necessary for defense against some biotrophic pathogens, and prevent pathogen infection by inducing stomatal closure (Ton and Mauch-Mani, 2004; Adie et al., 2007). Like ABA, polyamines were also shown to interact with JA and ET signaling, thereby enhancing plant susceptibility to pathogens (Anderson et al., 2004). The over accumulation of spermidine in transgenic lines of tomato was shown to be associated with impaired function of ET in induced defense responses, thereby increasing the susceptibility of fruit to *B. cinerea* (Nambeesan et al., 2012).

### Inhibition of Polyamine Oxidation Attenuates Defense Responses and Increases Susceptibility of Berries to *B. cinerea*

Pharmacological experiments with appropriate CuAO and PAO inhibitors prior to osmotic stress showed that inhibition of polyamine oxidation greatly enhanced polyamine levels and reduced the expression of defense genes and *NCED2*, as well as the amounts of resveratrol and ε-viniferin to different extents. These effects fit with the observations that the high level of free polyamines induced by osmotic stress in berries is correlated with high sensitivity to *B. cinerea* infection. Different studies showed that upregulation of polyamine oxidation is an important site of metabolic regulation involved in water stress tolerance or in resistance mechanisms toward pathogens (Marina et al., 2008; Moschou et al., 2008, 2012; Alcázar et al., 2010). Our data support the idea that both CuAO and PAO pathways could be required for an optimal level of polyamines, and subsequently for immune response in ripe berries. This is also in accordance with other previous findings (Takahashi et al., 2004; Marina et al., 2008), showing that inhibition of CuAO and PAO repressed the expression of defense-related genes.

The reduced expression of *NCED2* involved in ABA synthesis by CuAO inhibitor and/or osmotic stress suggests a relationship between polyamine oxidation and ABA synthesis under osmotic stress conditions. This is consistent with the amplification of symptoms of gray mold in berries by CuAO and PAO inhibitors prior to osmotic stress. These data highlight the role of polyamine titers in controlling defense responses and susceptibility of ripe berries to *B. cinerea*. Hydrogen peroxide derived from polyamine oxidation (Rea et al., 2002; Walters, 2003) and the balance between polyamine and ET synthesis (Nambeesan et al., 2012) may also be involved a major regulators of defense process.

**FIGURE 8:**
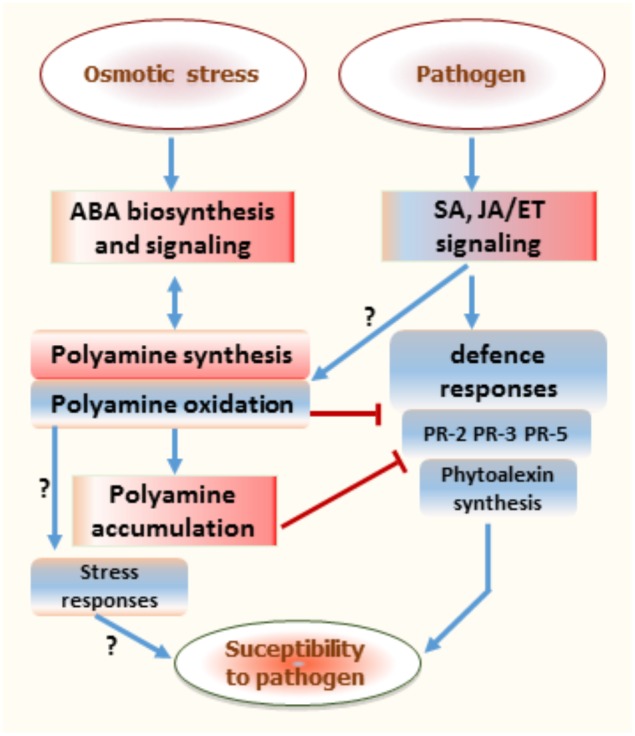
Proposed schematic diagram illustrating the role of polyamine status in regulating the interaction between osmotic stress and subsequent pathogen stress responses. Red color boxes indicate upregulation and blue boxes show downregulation. Blue arrows show induction or positive regulation, while red bars show inhibition or repression of response. ABA, abscisic acid; ET, ethylene; JA, jasmonic acid; SA, salicylic acid; PR, pathogenesis-related.

Overall, exposure of grapevine berries to osmotic stress prior pathogen infection may result in a complex interaction leading to enhanced disease susceptibility (**Figure 8**). Data from this study may suggest that osmotic stress or ABA slow down the expression of defense responses and then facilitate gray mold in ripe berries. The combined osmotic stress and *B. cinerea* challenge primed berries for enhanced polyamine accumulation, which is in part related to weak activation of their oxidation. This emphasizes the importance of polyamine level or their oxidative pathways as critical regulators of both osmotic stress signaling and triggered defense responses against pathogen. Under osmotic stress polyamines can interact with ABA synthesis and interfere with defense responses in grapevine berries, especially after pathogen infection. It is hypothesized that polyamine oxidation can benefit grapevine fitness by providing the trade-off between osmotic stress response and disease susceptibility.

## Significance Statement

Osmotic stress and ABA affect immune response in grape berries, while polyamine level may have a role in weakening plant defense and resistance to the necrotrophic pathogen *B. cinerea*.

## Author Contributions

SH and SV performed most of the experiments. PT-A provided substantial help in experiments. AA and SH designed the research, wrote the manuscript with contributions, and discussion from all the co-authors. All authors read and approved the final manuscript.

## Conflict of Interest Statement

The authors declare that the research was conducted in the absence of any commercial or financial relationships that could be construed as a potential conflict of interest.
