# Supplementary material for: Osmotic Stress and ABA Affect Immune Response and Susceptibility of Grapevine Berries to Gray Mold by Priming Polyamine Accumulation
Source: Front Plant Sci. 2018 Jul 11;9:1010. doi: 10.3389/fpls.2018.01010 (PMC6050403; doi:10.3389/fpls.2018.01010)
Supplement: Supplementary file 1 [file Table_1.DOCX]

**Supplementary Table S1**: Primer sequences of genes analyzed by RT-PCR

| **Gene** | **Accession number** | **Forward Primer (5’-3’)** | **Reverse primer (5’-3’)** |
| --- | --- | --- | --- |
| *STS* | NM_001281117.1 | AGGAAGCAGCATTGAAGGCTC | TGCACCAGGCATTTCTACACC |
| *PR2* | NM_001280967.1 | TCAATGGCTGCAATGGTGC | CGGTCGATGTTGCGAGATTTA |
| *PR3* | NM_001281244.1 | TCGAATGCGATGGTGGAAA | TCCCCTGTCGAAACACCAAG |
| *PR5* | NM_001281202.1 | CCTAACACCTTAGCCGAATTCGC | GGCCATAGGCACATTAAATCCATC |
| *NCED2* | XM_003632982.1 | CTCTTGGCCATGTCGGAAGA | CGGAGCTGCTTGTCGAAGTC |
| *EF1α* | XM_002284888.1 | AACCAAAATATCCGGAGTAAAAGA | GAACTGGGTGCTTGATAGGC |
| *60RSP* | XM_002270599.1 | ATCTACCTCAAGCTCCTAGTC | CAATCTTGTCCTCCTTTCCT |
